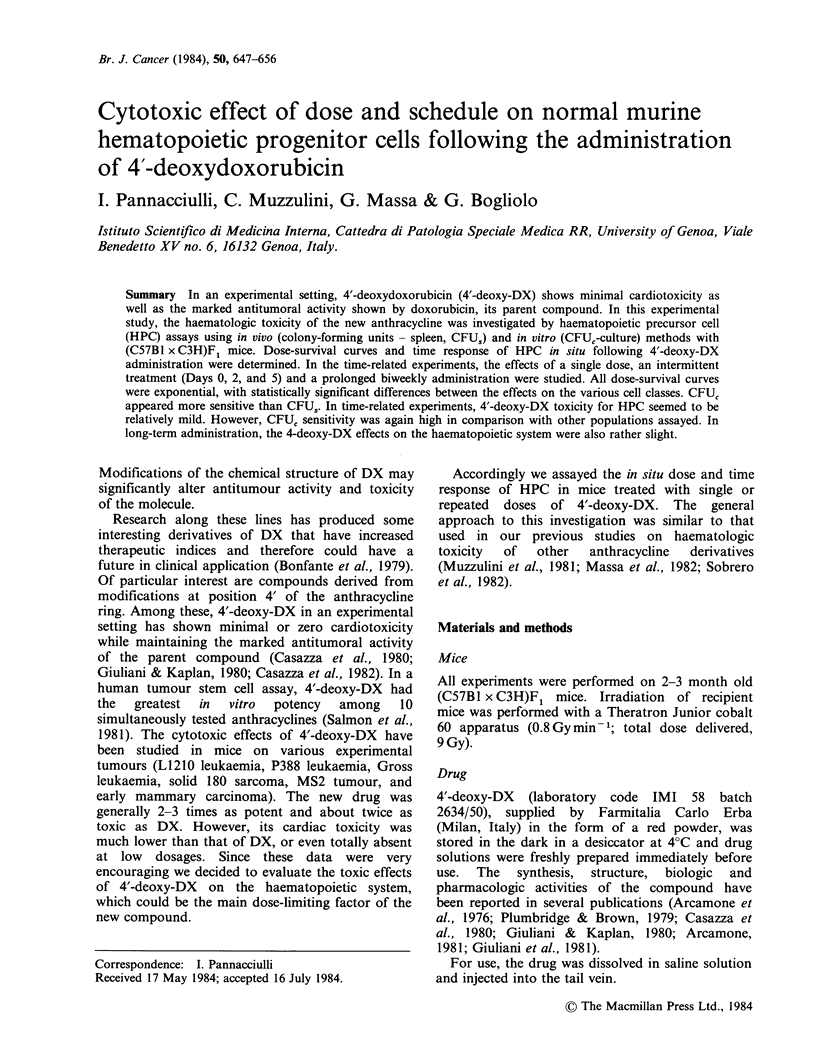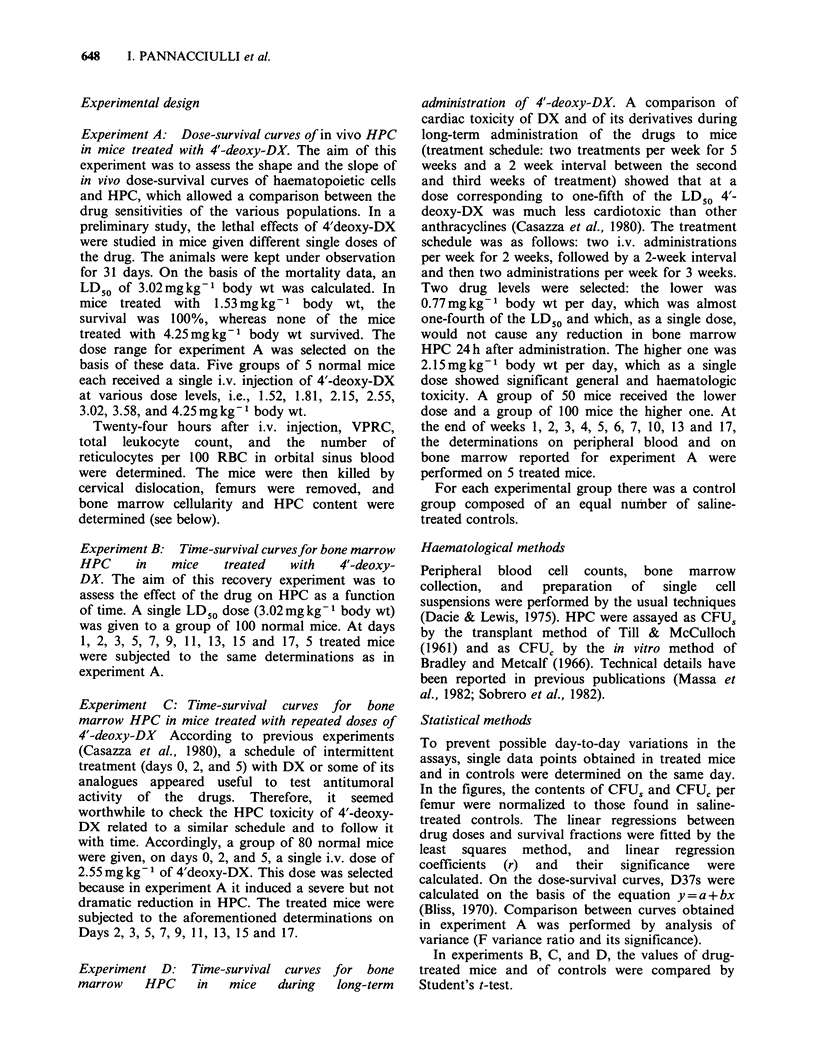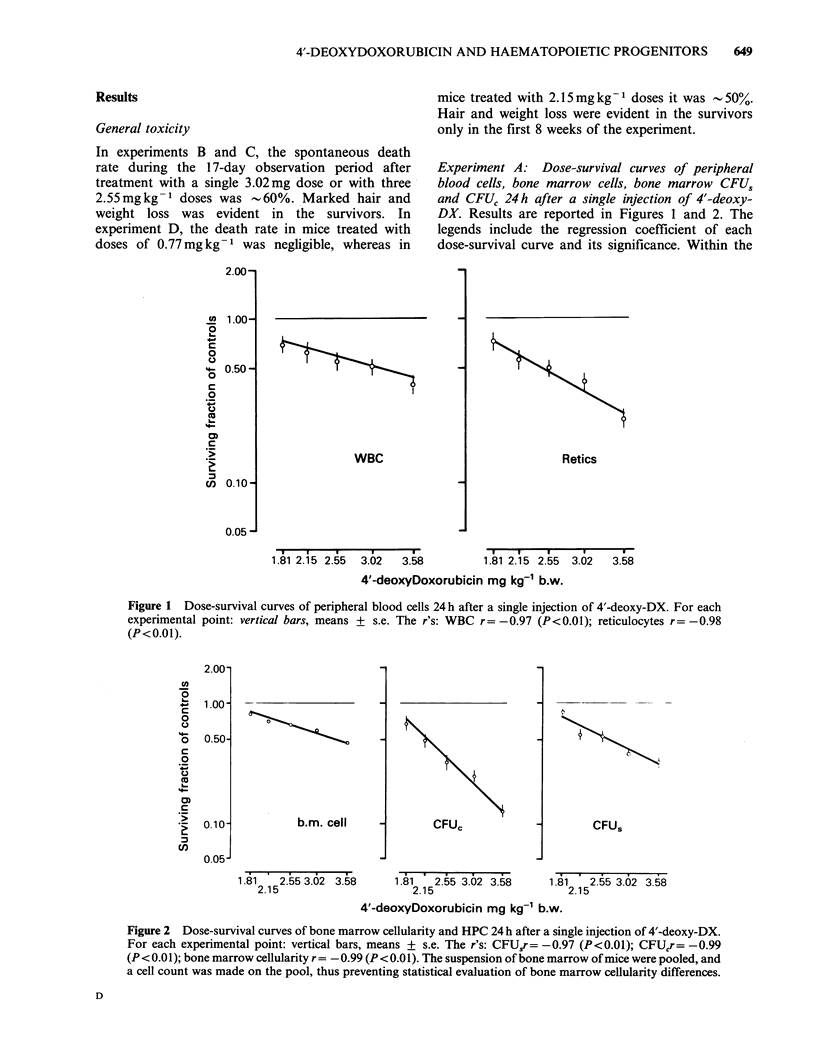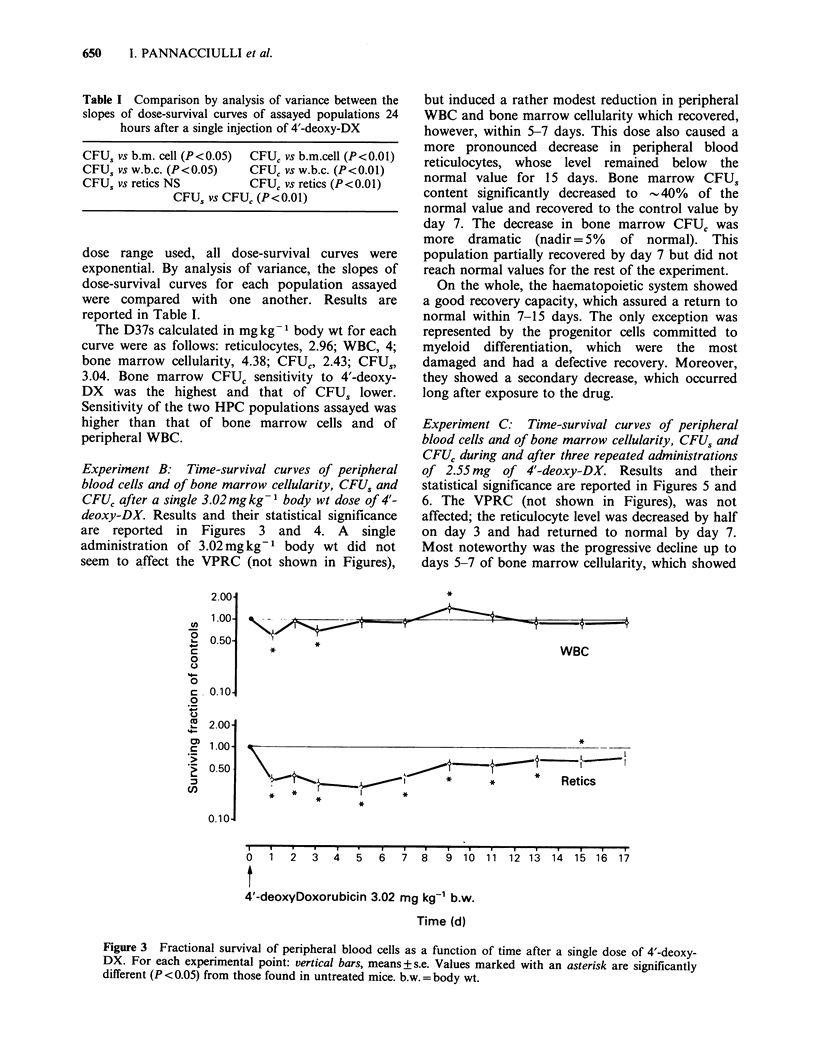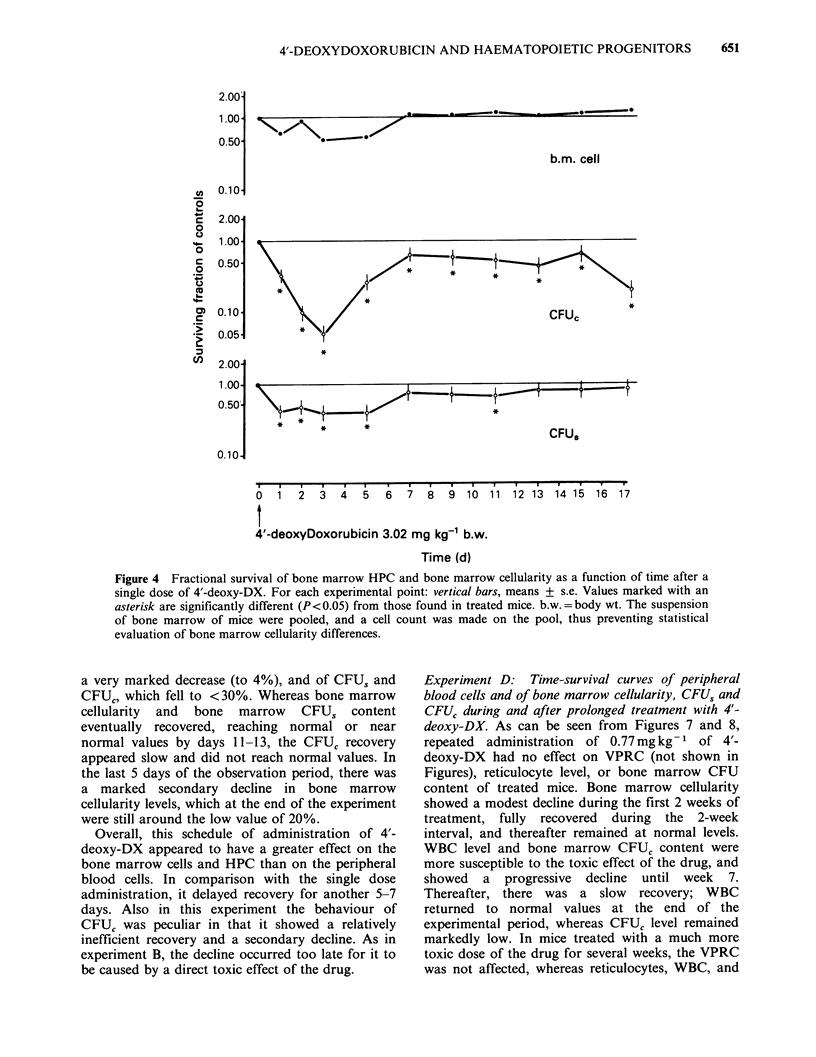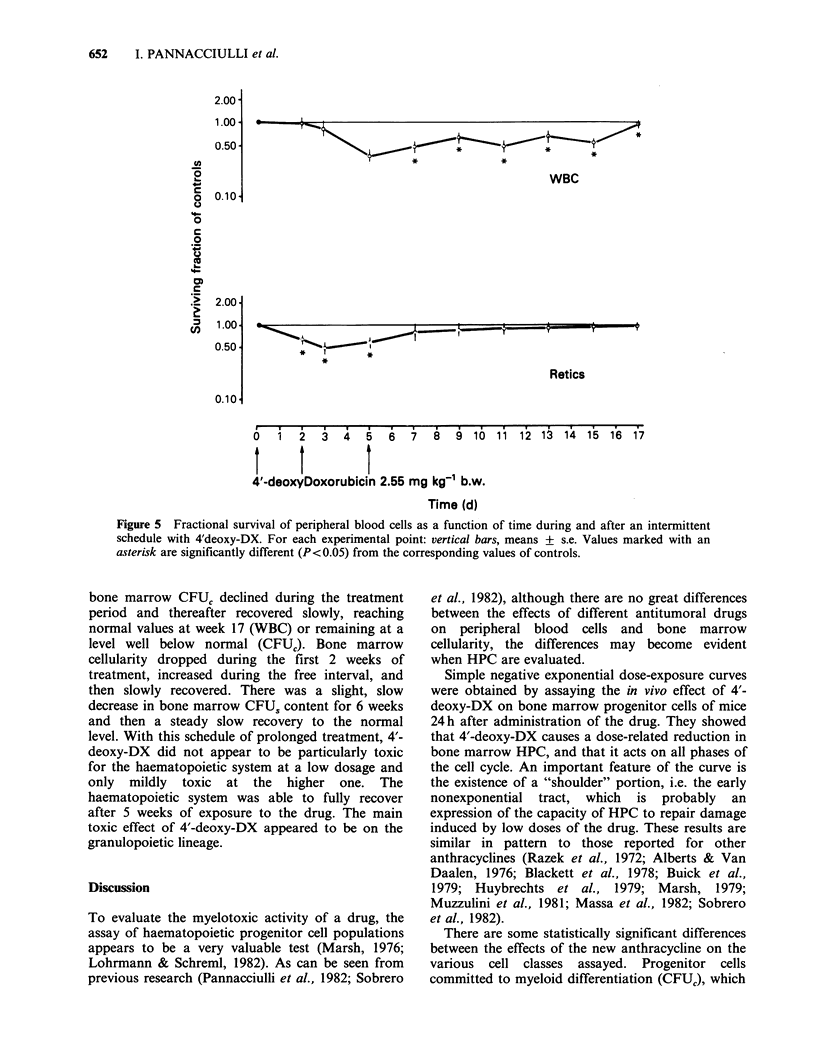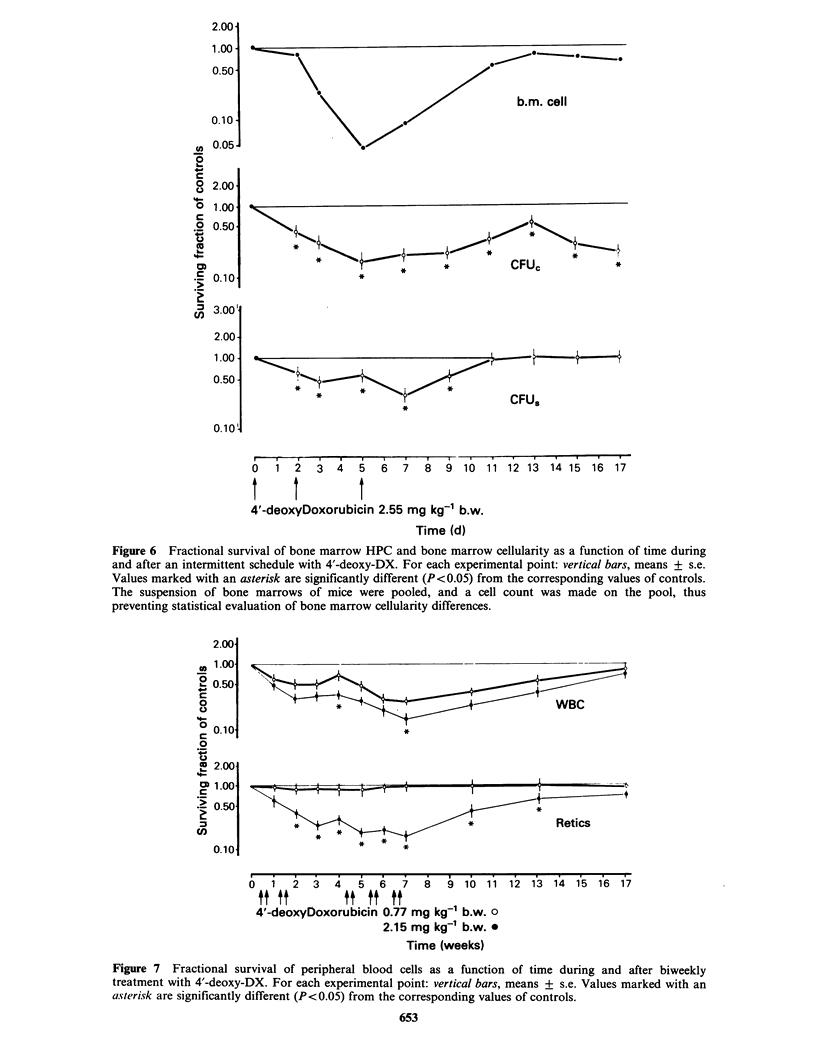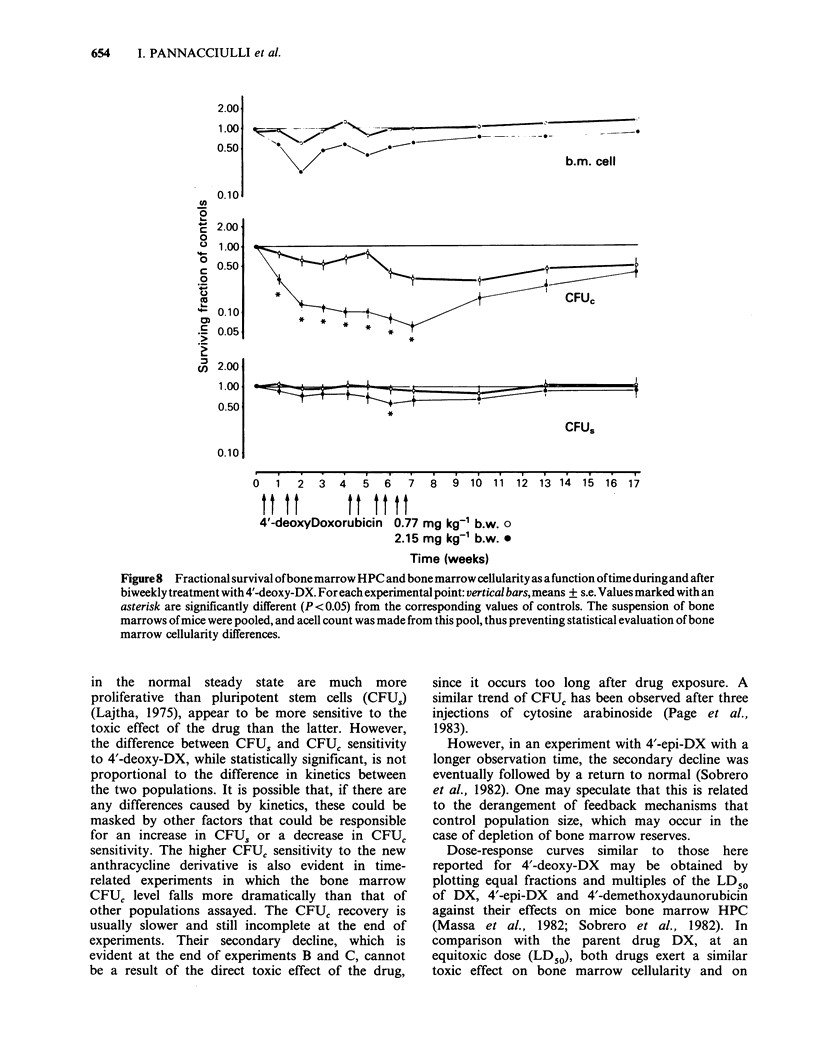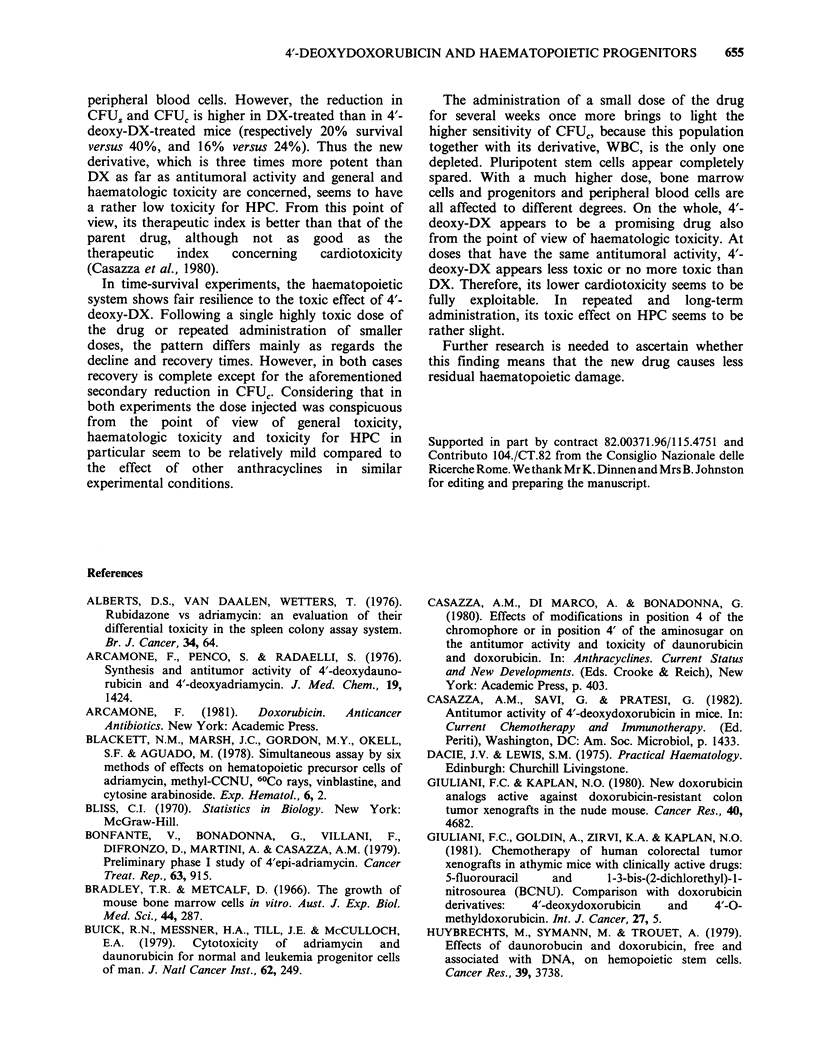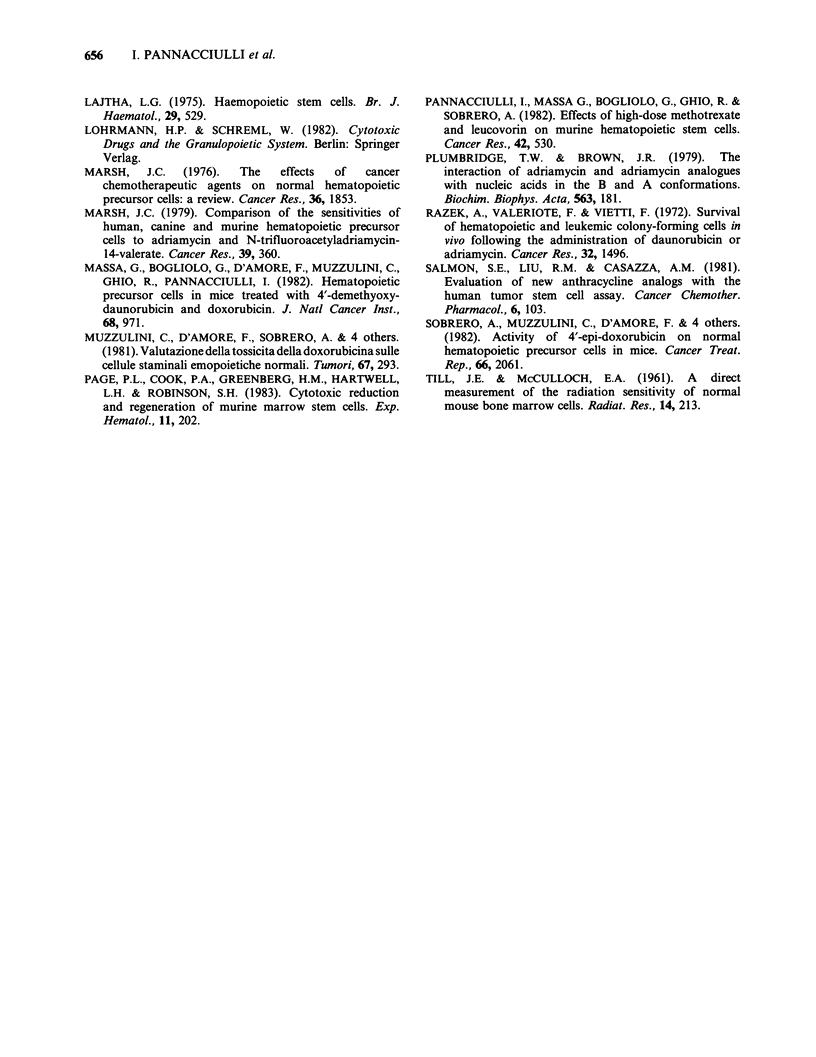# Cytotoxic effect of dose and schedule on normal murine hematopoietic progenitor cells following the administration of 4'-deoxydoxorubicin.

**DOI:** 10.1038/bjc.1984.231

**Published:** 1984-11

**Authors:** I. Pannacciulli, C. Muzzulini, G. Massa, G. Bogliolo

## Abstract

In an experimental setting, 4'-deoxydoxorubicin (4'-deoxy-DX) shows minimal cardiotoxicity as well as the marked antitumoral activity shown by doxorubicin, its parent compound. In this experimental study, the haematologic toxicity of the new anthracycline was investigated by haematopoietic precursor cell (HPC) assays using in vivo (colony-forming units - spleen, CFUs) and in vitro (CFUc-culture) methods with (C57B1 X C3H)F1 mice. Dose-survival curves and time response of HPC in situ following 4'-deoxy-DX administration were determined. In the time-related experiments, the effects of a single dose, an intermittent treatment (Days 0, 2, and 5) and a prolonged biweekly administration were studied. All dose-survival curves were exponential, with statistically significant differences between the effects on the various cell classes. CFUc appeared more sensitive than CFUs. In time-related experiments, 4'-deoxy-DX toxicity for HPC seemed to be relatively mild. However, CFUc sensitivity was again high in comparison with other populations assayed. In long-term administration, the 4-deoxy-DX effects on the haematopoietic system were also rather slight.


*Br. J. Cancer* (1984), **50**, 647–656

### Cytotoxic effect of dose and schedule on normal murine hematopoietic progenitor cells following the administration of 4′-deoxydoxorubicin

I. Pannacciulli, C. Muzzulini, G. Massa & G. Bogliolo

*Istituto Scientifico di Medicina Interna, Cattedra di Patologia Speciale Medica RR, University of Genoa, Viale Benedetto XV no. 6, 16132 Genoa, Italy.*

**Summary** In an experimental setting, 4′-deoxydoxorubicin (4′-deoxy-DX) shows minimal cardiotoxicity as well as the marked antitumoral activity shown by doxorubicin, its parent compound. In this experimental study, the haematologic toxicity of the new anthracycline was investigated by haematopoietic precursor cell (HPC) assays using *in vivo* (colony-forming units – spleen, $CFU_s$) and *in vitro* ($CFU_c$-culture) methods with $(C57B1 \times C3H)F_1$ mice. Dose-survival curves and time response of HPC *in situ* following 4′-deoxy-DX administration were determined. In the time-related experiments, the effects of a single dose, an intermittent treatment (Days 0, 2, and 5) and a prolonged biweekly administration were studied. All dose-survival curves were exponential, with statistically significant differences between the effects on the various cell classes. $CFU_c$ appeared more sensitive than $CFU_s$. In time-related experiments, 4′-deoxy-DX toxicity for HPC seemed to be relatively mild. However, $CFU_c$ sensitivity was again high in comparison with other populations assayed. In long-term administration, the 4-deoxy-DX effects on the haematopoietic system were also rather slight.

Modifications of the chemical structure of DX may significantly alter antitumour activity and toxicity of the molecule.

Research along these lines has produced some interesting derivatives of DX that have increased therapeutic indices and therefore could have a future in clinical application (Bonfante *et al.*, 1979). Of particular interest are compounds derived from modifications at position 4′ of the anthracycline ring. Among these, 4′-deoxy-DX in an experimental setting has shown minimal or zero cardiotoxicity while maintaining the marked antitumoral activity of the parent compound (Casazza *et al.*, 1980; Giuliani & Kaplan, 1980; Casazza *et al.*, 1982). In a human tumour stem cell assay, 4′-deoxy-DX had the greatest *in vitro* potency among 10 simultaneously tested anthracyclines (Salmon *et al.*, 1981). The cytotoxic effects of 4′-deoxy-DX have been studied in mice on various experimental tumours (L1210 leukaemia, P388 leukaemia, Gross leukaemia, solid 180 sarcoma, MS2 tumour, and early mammary carcinoma). The new drug was generally 2–3 times as potent and about twice as toxic as DX. However, its cardiac toxicity was much lower than that of DX, or even totally absent at low dosages. Since these data were very encouraging we decided to evaluate the toxic effects of 4′-deoxy-DX on the haematopoietic system, which could be the main dose-limiting factor of the new compound.

Correspondence: I. Pannacciulli
Received 17 May 1984; accepted 16 July 1984.

Accordingly we assayed the *in situ* dose and time response of HPC in mice treated with single or repeated doses of 4′-deoxy-DX. The general approach to this investigation was similar to that used in our previous studies on haematologic toxicity of other anthracycline derivatives (Muzzulini *et al.*, 1981; Massa *et al.*, 1982; Sobrero *et al.*, 1982).

### Materials and methods

### *Mice*

All experiments were performed on 2–3 month old $(C57B1 \times C3H)F_1$ mice. Irradiation of recipient mice was performed with a Theratron Junior cobalt 60 apparatus ($0.8\,Gy\,min^{-1}$; total dose delivered, 9 Gy).

### *Drug*

4′-deoxy-DX (laboratory code IMI 58 batch 2634/50), supplied by Farmitalia Carlo Erba (Milan, Italy) in the form of a red powder, was stored in the dark in a desiccator at 4°C and drug solutions were freshly prepared immediately before use. The synthesis, structure, biologic and pharmacologic activities of the compound have been reported in several publications (Arcamone *et al.*, 1976; Plumbridge & Brown, 1979; Casazza *et al.*, 1980; Giuliani & Kaplan, 1980; Arcamone, 1981; Giuliani *et al.*, 1981).

For use, the drug was dissolved in saline solution and injected into the tail vein.

© The Macmillan Press Ltd., 1984

### Experimental design

*Experiment A: Dose-survival curves of* in vivo *HPC in mice treated with 4'-deoxy-DX.* The aim of this experiment was to assess the shape and the slope of *in vivo* dose-survival curves of haematopoietic cells and HPC, which allowed a comparison between the drug sensitivities of the various populations. In a preliminary study, the lethal effects of 4'deoxy-DX were studied in mice given different single doses of the drug. The animals were kept under observation for 31 days. On the basis of the mortality data, an $LD_{50}$ of $3.02\,mg\,kg^{-1}$ body wt was calculated. In mice treated with $1.53\,mg\,kg^{-1}$ body wt, the survival was 100%, whereas none of the mice treated with $4.25\,mg\,kg^{-1}$ body wt survived. The dose range for experiment A was selected on the basis of these data. Five groups of 5 normal mice each received a single i.v. injection of 4'-deoxy-DX at various dose levels, i.e., 1.52, 1.81, 2.15, 2.55, 3.02, 3.58, and $4.25\,mg\,kg^{-1}$ body wt.

Twenty-four hours after i.v. injection, VPRC, total leukocyte count, and the number of reticulocytes per 100 RBC in orbital sinus blood were determined. The mice were then killed by cervical dislocation, femurs were removed, and bone marrow cellularity and HPC content were determined (see below).

*Experiment B: Time-survival curves for bone marrow HPC in mice treated with 4'-deoxy-DX.* The aim of this recovery experiment was to assess the effect of the drug on HPC as a function of time. A single $LD_{50}$ dose ($3.02\,mg\,kg^{-1}$ body wt) was given to a group of 100 normal mice. At days 1, 2, 3, 5, 7, 9, 11, 13, 15 and 17, 5 treated mice were subjected to the same determinations as in experiment A.

*Experiment C: Time-survival curves for bone marrow HPC in mice treated with repeated doses of 4'-deoxy-DX* According to previous experiments (Casazza *et al.*, 1980), a schedule of intermittent treatment (days 0, 2, and 5) with DX or some of its analogues appeared useful to test antitumoral activity of the drugs. Therefore, it seemed worthwhile to check the HPC toxicity of 4'-deoxy-DX related to a similar schedule and to follow it with time. Accordingly, a group of 80 normal mice were given, on days 0, 2, and 5, a single i.v. dose of $2.55\,mg\,kg^{-1}$ of 4'deoxy-DX. This dose was selected because in experiment A it induced a severe but not dramatic reduction in HPC. The treated mice were subjected to the aforementioned determinations on Days 2, 3, 5, 7, 9, 11, 13, 15 and 17.

*Experiment D: Time-survival curves for bone marrow HPC in mice during long-term administration of 4'-deoxy-DX.* A comparison of cardiac toxicity of DX and of its derivatives during long-term administration of the drugs to mice (treatment schedule: two treatments per week for 5 weeks and a 2 week interval between the second and third weeks of treatment) showed that at a dose corresponding to one-fifth of the $LD_{50}$ 4'-deoxy-DX was much less cardiotoxic than other anthracyclines (Casazza *et al.*, 1980). The treatment schedule was as follows: two i.v. administrations per week for 2 weeks, followed by a 2-week interval and then two administrations per week for 3 weeks. Two drug levels were selected: the lower was $0.77\,mg\,kg^{-1}$ body wt per day, which was almost one-fourth of the $LD_{50}$ and which, as a single dose, would not cause any reduction in bone marrow HPC 24 h after administration. The higher one was $2.15\,mg\,kg^{-1}$ body wt per day, which as a single dose showed significant general and haematologic toxicity. A group of 50 mice received the lower dose and a group of 100 mice the higher one. At the end of weeks 1, 2, 3, 4, 5, 6, 7, 10, 13 and 17, the determinations on peripheral blood and on bone marrow reported for experiment A were performed on 5 treated mice.

For each experimental group there was a control group composed of an equal number of saline-treated controls.

### Haematological methods

Peripheral blood cell counts, bone marrow collection, and preparation of single cell suspensions were performed by the usual techniques (Dacie & Lewis, 1975). HPC were assayed as $CFU_s$ by the transplant method of Till & McCulloch (1961) and as $CFU_c$ by the *in vitro* method of Bradley and Metcalf (1966). Technical details have been reported in previous publications (Massa *et al.*, 1982; Sobrero *et al.*, 1982).

### Statistical methods

To prevent possible day-to-day variations in the assays, single data points obtained in treated mice and in controls were determined on the same day. In the figures, the contents of $CFU_s$ and $CFU_c$ per femur were normalized to those found in saline-treated controls. The linear regressions between drug doses and survival fractions were fitted by the least squares method, and linear regression coefficients ($r$) and their significance were calculated. On the dose-survival curves, D37s were calculated on the basis of the equation $y = a + bx$ (Bliss, 1970). Comparison between curves obtained in experiment A was performed by analysis of variance (F variance ratio and its significance).

In experiments B, C, and D, the values of drug-treated mice and of controls were compared by Student's $t$-test.

### Results

### *General toxicity*

In experiments B and C, the spontaneous death rate during the 17-day observation period after treatment with a single 3.02 mg dose or with three 2.55 mg $kg^{-1}$ doses was ~60%. Marked hair and weight loss was evident in the survivors. In experiment D, the death rate in mice treated with doses of 0.77 mg $kg^{-1}$ was negligible, whereas in mice treated with 2.15 mg $kg^{-1}$ doses it was ~50%. Hair and weight loss were evident in the survivors only in the first 8 weeks of the experiment.

*Experiment A: Dose-survival curves of peripheral blood cells, bone marrow cells, bone marrow $CFU_s$ and $CFU_c$ 24 h after a single injection of 4'-deoxy-DX.* Results are reported in Figures 1 and 2. The legends include the regression coefficient of each dose-survival curve and its significance. Within the

**Figure 1** Dose-survival curves of peripheral blood cells 24 h after a single injection of 4'-deoxy-DX. For each experimental point: *vertical bars*, means ± s.e. The *r*'s: WBC $r=-0.97$ ($P<0.01$); reticulocytes $r=-0.98$ ($P<0.01$).

**Figure 2** Dose-survival curves of bone marrow cellularity and HPC 24 h after a single injection of 4'-deoxy-DX. For each experimental point: vertical bars, means ± s.e. The *r*'s: $CFU_s$ $r=-0.97$ ($P<0.01$); $CFU_c$ $r=-0.99$ ($P<0.01$); bone marrow cellularity $r=-0.99$ ($P<0.01$). The suspension of bone marrow of mice were pooled, and a cell count was made on the pool, thus preventing statistical evaluation of bone marrow cellularity differences.

**Table I** Comparison by analysis of variance between the slopes of dose-survival curves of assayed populations 24 hours after a single injection of 4′-deoxy-DX

| | |
|---|---|
| $CFU_s$ *vs* b.m. cell ($P<0.05$) | $CFU_c$ *vs* b.m.cell ($P<0.01$) |
| $CFU_s$ *vs* w.b.c. ($P<0.05$) | $CFU_c$ *vs* w.b.c. ($P<0.01$) |
| $CFU_s$ *vs* retics NS | $CFU_c$ *vs* retics ($P<0.01$) |
| $CFU_s$ *vs* $CFU_c$ ($P<0.01$) | |

dose range used, all dose-survival curves were exponential. By analysis of variance, the slopes of dose-survival curves for each population assayed were compared with one another. Results are reported in Table I.

The D37s calculated in mg kg$^{-1}$ body wt for each curve were as follows: reticulocytes, 2.96; WBC, 4; bone marrow cellularity, 4.38; $CFU_c$, 2.43; $CFU_s$, 3.04. Bone marrow $CFU_c$ sensitivity to 4′-deoxy-DX was the highest and that of $CFU_s$ lower. Sensitivity of the two HPC populations assayed was higher than that of bone marrow cells and of peripheral WBC.

*Experiment B: Time-survival curves of peripheral blood cells and of bone marrow cellularity, $CFU_s$ and $CFU_c$ after a single 3.02 mg kg$^{-1}$ body wt dose of 4′-deoxy-DX.* Results and their statistical significance are reported in Figures 3 and 4. A single administration of 3.02 mg kg$^{-1}$ body wt did not seem to affect the VPRC (not shown in Figures), but induced a rather modest reduction in peripheral WBC and bone marrow cellularity which recovered, however, within 5–7 days. This dose also caused a more pronounced decrease in peripheral blood reticulocytes, whose level remained below the normal value for 15 days. Bone marrow $CFU_s$ content significantly decreased to ~40% of the normal value and recovered to the control value by day 7. The decrease in bone marrow $CFU_c$ was more dramatic (nadir = 5% of normal). This population partially recovered by day 7 but did not reach normal values for the rest of the experiment.

On the whole, the haematopoietic system showed a good recovery capacity, which assured a return to normal within 7–15 days. The only exception was represented by the progenitor cells committed to myeloid differentiation, which were the most damaged and had a defective recovery. Moreover, they showed a secondary decrease, which occurred long after exposure to the drug.

*Experiment C: Time-survival curves of peripheral blood cells and of bone marrow cellularity, $CFU_s$ and $CFU_c$ during and after three repeated administrations of 2.55 mg of 4′-deoxy-DX.* Results and their statistical significance are reported in Figures 5 and 6. The VPRC (not shown in Figures), was not affected; the reticulocyte level was decreased by half on day 3 and had returned to normal by day 7. Most noteworthy was the progressive decline up to days 5–7 of bone marrow cellularity, which showed

**Figure 3** Fractional survival of peripheral blood cells as a function of time after a single dose of 4′-deoxy-DX. For each experimental point: *vertical bars*, means ± s.e. Values marked with an *asterisk* are significantly different ($P<0.05$) from those found in untreated mice. b.w. = body wt.

**Figure 4** Fractional survival of bone marrow HPC and bone marrow cellularity as a function of time after a single dose of 4′-deoxy-DX. For each experimental point: *vertical bars*, means ± s.e. Values marked with an *asterisk* are significantly different ($P<0.05$) from those found in treated mice. b.w. = body wt. The suspension of bone marrow of mice were pooled, and a cell count was made on the pool, thus preventing statistical evaluation of bone marrow cellularity differences.

a very marked decrease (to 4%), and of $CFU_s$ and $CFU_c$, which fell to <30%. Whereas bone marrow cellularity and bone marrow $CFU_s$ content eventually recovered, reaching normal or near normal values by days 11–13, the $CFU_c$ recovery appeared slow and did not reach normal values. In the last 5 days of the observation period, there was a marked secondary decline in bone marrow cellularity levels, which at the end of the experiment were still around the low value of 20%.

Overall, this schedule of administration of 4′-deoxy-DX appeared to have a greater effect on the bone marrow cells and HPC than on the peripheral blood cells. In comparison with the single dose administration, it delayed recovery for another 5–7 days. Also in this experiment the behaviour of $CFU_c$ was peculiar in that it showed a relatively inefficient recovery and a secondary decline. As in experiment B, the decline occurred too late for it to be caused by a direct toxic effect of the drug.

*Experiment D: Time-survival curves of peripheral blood cells and of bone marrow cellularity, $CFU_s$ and $CFU_c$ during and after prolonged treatment with 4′-deoxy-DX.* As can be seen from Figures 7 and 8, repeated administration of $0.77\,mg\,kg^{-1}$ of 4′-deoxy-DX had no effect on VPRC (not shown in Figures), reticulocyte level, or bone marrow CFU content of treated mice. Bone marrow cellularity showed a modest decline during the first 2 weeks of treatment, fully recovered during the 2-week interval, and thereafter remained at normal levels. WBC level and bone marrow $CFU_c$ content were more susceptible to the toxic effect of the drug, and showed a progressive decline until week 7. Thereafter, there was a slow recovery; WBC returned to normal values at the end of the experimental period, whereas $CFU_c$ level remained markedly low. In mice treated with a much more toxic dose of the drug for several weeks, the VPRC was not affected, whereas reticulocytes, WBC, and

**Figure 5** Fractional survival of peripheral blood cells as a function of time during and after an intermittent schedule with 4'deoxy-DX. For each experimental point: *vertical bars*, means ± s.e. Values marked with an *asterisk* are significantly different ($P<0.05$) from the corresponding values of controls.

bone marrow $CFU_c$ declined during the treatment period and thereafter recovered slowly, reaching normal values at week 17 (WBC) or remaining at a level well below normal ($CFU_c$). Bone marrow cellularity dropped during the first 2 weeks of treatment, increased during the free interval, and then slowly recovered. There was a slight, slow decrease in bone marrow $CFU_s$ content for 6 weeks and then a steady slow recovery to the normal level. With this schedule of prolonged treatment, 4'-deoxy-DX did not appear to be particularly toxic for the haematopoietic system at a low dosage and only mildly toxic at the higher one. The haematopoietic system was able to fully recover after 5 weeks of exposure to the drug. The main toxic effect of 4'-deoxy-DX appeared to be on the granulopoietic lineage.

### Discussion

To evaluate the myelotoxic activity of a drug, the assay of haematopoietic progenitor cell populations appears to be a very valuable test (Marsh, 1976; Lohrmann & Schreml, 1982). As can be seen from previous research (Pannacciulli *et al.*, 1982; Sobrero *et al.*, 1982), although there are no great differences between the effects of different antitumoral drugs on peripheral blood cells and bone marrow cellularity, the differences may become evident when HPC are evaluated.

Simple negative exponential dose-exposure curves were obtained by assaying the *in vivo* effect of 4'-deoxy-DX on bone marrow progenitor cells of mice 24 h after administration of the drug. They showed that 4'-deoxy-DX causes a dose-related reduction in bone marrow HPC, and that it acts on all phases of the cell cycle. An important feature of the curve is the existence of a "shoulder" portion, i.e. the early nonexponential tract, which is probably an expression of the capacity of HPC to repair damage induced by low doses of the drug. These results are similar in pattern to those reported for other anthracyclines (Razek *et al.*, 1972; Alberts & Van Daalen, 1976; Blackett *et al.*, 1978; Buick *et al.*, 1979; Huybrechts *et al.*, 1979; Marsh, 1979; Muzzulini *et al.*, 1981; Massa *et al.*, 1982; Sobrero *et al.*, 1982).

There are some statistically significant differences between the effects of the new anthracycline on the various cell classes assayed. Progenitor cells committed to myeloid differentiation ($CFU_c$), which

**Figure 6** Fractional survival of bone marrow HPC and bone marrow cellularity as a function of time during and after an intermittent schedule with 4′-deoxy-DX. For each experimental point: *vertical bars*, means ± s.e. Values marked with an *asterisk* are significantly different ($P<0.05$) from the corresponding values of controls. The suspension of bone marrows of mice were pooled, and a cell count was made on the pool, thus preventing statistical evaluation of bone marrow cellularity differences.

**Figure 7** Fractional survival of peripheral blood cells as a function of time during and after biweekly treatment with 4′-deoxy-DX. For each experimental point: *vertical bars*, means ± s.e. Values marked with an *asterisk* are significantly different ($P<0.05$) from the corresponding values of controls.

**Figure 8** Fractional survival of bone marrow HPC and bone marrow cellularity as a function of time during and after biweekly treatment with 4′-deoxy-DX. For each experimental point: *vertical bars*, means ± s.e. Values marked with an *asterisk* are significantly different ($P<0.05$) from the corresponding values of controls. The suspension of bone marrows of mice were pooled, and acell count was made from this pool, thus preventing statistical evaluation of bone marrow cellularity differences.

in the normal steady state are much more proliferative than pluripotent stem cells (CFU$_s$) (Lajtha, 1975), appear to be more sensitive to the toxic effect of the drug than the latter. However, the difference between CFU$_s$ and CFU$_c$ sensitivity to 4′-deoxy-DX, while statistically significant, is not proportional to the difference in kinetics between the two populations. It is possible that, if there are any differences caused by kinetics, these could be masked by other factors that could be responsible for an increase in CFU$_s$ or a decrease in CFU$_c$ sensitivity. The higher CFU$_c$ sensitivity to the new anthracycline derivative is also evident in time-related experiments in which the bone marrow CFU$_c$ level falls more dramatically than that of other populations assayed. The CFU$_c$ recovery is usually slower and still incomplete at the end of experiments. Their secondary decline, which is evident at the end of experiments B and C, cannot be a result of the direct toxic effect of the drug, since it occurs too long after drug exposure. A similar trend of CFU$_c$ has been observed after three injections of cytosine arabinoside (Page *et al.*, 1983).

However, in an experiment with 4′-epi-DX with a longer observation time, the secondary decline was eventually followed by a return to normal (Sobrero *et al.*, 1982). One may speculate that this is related to the derangement of feedback mechanisms that control population size, which may occur in the case of depletion of bone marrow reserves.

Dose-response curves similar to those here reported for 4′-deoxy-DX may be obtained by plotting equal fractions and multiples of the $LD_{50}$ of DX, 4′-epi-DX and 4′-demethoxydaunorubicin against their effects on mice bone marrow HPC (Massa *et al.*, 1982; Sobrero *et al.*, 1982). In comparison with the parent drug DX, at an equitoxic dose ($LD_{50}$), both drugs exert a similar toxic effect on bone marrow cellularity and on peripheral blood cells. However, the reduction in $CFU_s$ and $CFU_c$ is higher in DX-treated than in 4′-deoxy-DX-treated mice (respectively 20% survival *versus* 40%, and 16% *versus* 24%). Thus the new derivative, which is three times more potent than DX as far as antitumoral activity and general and haematologic toxicity are concerned, seems to have a rather low toxicity for HPC. From this point of view, its therapeutic index is better than that of the parent drug, although not as good as the therapeutic index concerning cardiotoxicity (Casazza *et al.*, 1980).

In time-survival experiments, the haematopoietic system shows fair resilience to the toxic effect of 4′-deoxy-DX. Following a single highly toxic dose of the drug or repeated administration of smaller doses, the pattern differs mainly as regards the decline and recovery times. However, in both cases recovery is complete except for the aforementioned secondary reduction in $CFU_c$. Considering that in both experiments the dose injected was conspicuous from the point of view of general toxicity, haematologic toxicity and toxicity for HPC in particular seem to be relatively mild compared to the effect of other anthracyclines in similar experimental conditions.

The administration of a small dose of the drug for several weeks once more brings to light the higher sensitivity of $CFU_c$, because this population together with its derivative, WBC, is the only one depleted. Pluripotent stem cells appear completely spared. With a much higher dose, bone marrow cells and progenitors and peripheral blood cells are all affected to different degrees. On the whole, 4′-deoxy-DX appears to be a promising drug also from the point of view of haematologic toxicity. At doses that have the same antitumoral activity, 4′-deoxy-DX appears less toxic or no more toxic than DX. Therefore, its lower cardiotoxicity seems to be fully exploitable. In repeated and long-term administration, its toxic effect on HPC seems to be rather slight.

Further research is needed to ascertain whether this finding means that the new drug causes less residual haematopoietic damage.

Supported in part by contract 82.00371.96/115.4751 and Contributo 104./CT.82 from the Consiglio Nazionale delle Ricerche Rome. We thank Mr K. Dinnen and Mrs B. Johnston for editing and preparing the manuscript.

**References**

ALBERTS, D.S., VAN DAALEN, WETTERS, T. (1976). Rubidazone vs adriamycin: an evaluation of their differential toxicity in the spleen colony assay system. *Br. J. Cancer*, **34**, 64.

ARCAMONE, F., PENCO, S. & RADAELLI, S. (1976). Synthesis and antitumor activity of 4′-deoxydaunorubicin and 4′-deoxyadriamycin. *J. Med. Chem.*, **19**, 1424.

ARCAMONE, F. (1981). *Doxorubicin. Anticancer Antibiotics*. New York: Academic Press.

BLACKETT, N.M., MARSH, J.C., GORDON, M.Y., OKELL, S.F. & AGUADO, M. (1978). Simultaneous assay by six methods of effects on hematopoietic precursor cells of adriamycin, methyl-CCNU, $^{60}Co$ rays, vinblastine, and cytosine arabinoside. *Exp. Hematol.*, **6**, 2.

BLISS, C.I. (1970). *Statistics in Biology*. New York: McGraw-Hill.

BONFANTE, V., BONADONNA, G., VILLANI, F., DIFRONZO, D., MARTINI, A. & CASAZZA, A.M. (1979). Preliminary phase I study of 4′epi-adriamycin. *Cancer Treat. Rep.*, **63**, 915.

BRADLEY, T.R. & METCALF, D. (1966). The growth of mouse bone marrow cells *in vitro*. *Aust. J. Exp. Biol. Med. Sci.*, **44**, 287.

BUICK, R.N., MESSNER, H.A., TILL, J.E. & McCULLOCH, E.A. (1979). Cytotoxicity of adriamycin and daunorubicin for normal and leukemia progenitor cells of man. *J. Natl Cancer Inst.*, **62**, 249.

CASAZZA, A.M., DI MARCO, A. & BONADONNA, G. (1980). Effects of modifications in position 4 of the chromophore or in position 4′ of the aminosugar on the antitumor activity and toxicity of daunorubicin and doxorubicin. In: *Anthracyclines. Current Status and New Developments*. (Eds. Crooke & Reich), New York: Academic Press, p. 403.

CASAZZA, A.M., SAVI, G. & PRATESI, G. (1982). Antitumor activity of 4′-deoxydoxorubicin in mice. In: *Current Chemotherapy and Immunotherapy*. (Ed. Periti), Washington, DC: Am. Soc. Microbiol, p. 1433.

DACIE, J.V. & LEWIS, S.M. (1975). *Practical Haematology*. Edinburgh: Churchill Livingstone.

GIULIANI, F.C. & KAPLAN, N.O. (1980). New doxorubicin analogs active against doxorubicin-resistant colon tumor xenografts in the nude mouse. *Cancer Res.*, **40**, 4682.

GIULIANI, F.C., GOLDIN, A., ZIRVI, K.A. & KAPLAN, N.O. (1981). Chemotherapy of human colorectal tumor xenografts in athymic mice with clinically active drugs: 5-fluorouracil and 1-3-bis-(2-dichlorethyl)-1-nitrosourea (BCNU). Comparison with doxorubicin derivatives: 4′-deoxydoxorubicin and 4′-O-methyldoxorubicin. *Int. J. Cancer*, **27**, 5.

HUYBRECHTS, M., SYMANN, M. & TROUET, A. (1979). Effects of daunorobucin and doxorubicin, free and associated with DNA, on hemopoietic stem cells. *Cancer Res.*, **39**, 3738.

LAJTHA, L.G. (1975). Haemopoietic stem cells. *Br. J. Haematol.*, **29**, 529.

LOHRMANN, H.P. & SCHREML, W. (1982). *Cytotoxic Drugs and the Granulopoietic System*. Berlin: Springer Verlag.

MARSH, J.C. (1976). The effects of cancer chemotherapeutic agents on normal hematopoietic precursor cells: a review. *Cancer Res.*, **36**, 1853.

MARSH, J.C. (1979). Comparison of the sensitivities of human, canine and murine hematopoietic precursor cells to adriamycin and N-trifluoroacetyladriamycin-14-valerate. *Cancer Res.*, **39**, 360.

MASSA, G., BOGLIOLO, G., D'AMORE, F., MUZZULINI, C., GHIO, R., PANNACCIULLI, I. (1982). Hematopoietic precursor cells in mice treated with 4′-demethyoxy-daunorubicin and doxorubicin. *J. Natl Cancer Inst.*, **68**, 971.

MUZZULINI, C., D'AMORE, F., SOBRERO, A. & 4 others. (1981). Valutazione della tossicita della doxorubicina sulle cellule staminali emopoietiche normali. *Tumori*, **67**, 293.

PAGE, P.L., COOK, P.A., GREENBERG, H.M., HARTWELL, L.H. & ROBINSON, S.H. (1983). Cytotoxic reduction and regeneration of murine marrow stem cells. *Exp. Hematol.*, **11**, 202.

PANNACCIULLI, I., MASSA G., BOGLIOLO, G., GHIO, R. & SOBRERO, A. (1982). Effects of high-dose methotrexate and leucovorin on murine hematopoietic stem cells. *Cancer Res.*, **42**, 530.

PLUMBRIDGE, T.W. & BROWN, J.R. (1979). The interaction of adriamycin and adriamycin analogues with nucleic acids in the B and A conformations. *Biochim. Biophys. Acta*, **563**, 181.

RAZEK, A., VALERIOTE, F. & VIETTI, F. (1972). Survival of hematopoietic and leukemic colony-forming cells *in vivo* following the administration of daunorubicin or adriamycin. *Cancer Res.*, **32**, 1496.

SALMON, S.E., LIU, R.M. & CASAZZA, A.M. (1981). Evaluation of new anthracycline analogs with the human tumor stem cell assay. *Cancer Chemother. Pharmacol.*, **6**, 103.

SOBRERO, A., MUZZULINI, C., D'AMORE, F. & 4 others. (1982). Activity of 4′-epi-doxorubicin on normal hematopoietic precursor cells in mice. *Cancer Treat. Rep.*, **66**, 2061.

TILL, J.E. & MCCULLOCH, E.A. (1961). A direct measurement of the radiation sensitivity of normal mouse bone marrow cells. *Radiat. Res.*, **14**, 213.